# Improving assessment of procedural skills in health sciences education: a validation study of a rubrics system in neurophysiotherapy

**DOI:** 10.1186/s40359-024-01643-7

**Published:** 2024-03-14

**Authors:** Rafael Garcia-Ros, Maria-Arantzazu Ruescas-Nicolau, Natalia Cezón-Serrano, Cristina Flor-Rufino, Constanza San Martin-Valenzuela, M. Luz Sánchez-Sánchez

**Affiliations:** 1 Department of Developmental and Educational Psychology, Faculty of Psychology, University of Valencia, Blasco Ibáñez Av. no. 21, Valencia, 46010 Spain; 2 Neurophysiotherapy Teaching Innovation Group, Department of Physiotherapy, Faculty of Physiotherapy, University of Valencia, Gascó Oliag Street no. 5, Valencia, 46010 Spain; 3 Physiotherapy in Motion. Multispeciality Research Group (PTinMOTION), Department of Physiotherapy, Faculty of Physiotherapy, University of Valencia, Gascó Oliag Street no. 5, Valencia, 46010 Spain; 4 Department of Physiotherapy, Faculty of Physiotherapy, University of Valencia, Gascó Oliag Street no. 5, Valencia, 46010 Spain; 5 Research unit in Clinical biomechanics - UBIC, Department of Physiotherapy, Faculty of Physiotherapy, University of Valencia, Gascó Oliag Street no. 5, Valencia, 46010 Spain

**Keywords:** Rubrics, Formative assessment, Summative assessment, Validity, Reliability, Procedural skills, Physical therapy education

## Abstract

**Background:**

The development of procedural skills is essential in health sciences education. Rubrics can be useful for learning and assessing these skills. To this end, a set of rubrics were developed in case of neurophysiotherapy maneuvers for undergraduates. Although students found the rubrics to be valid and useful in previous courses, the analysis of the practical exam results showed the need to change them in order to improve their validity and reliability, especially when used for summative purposes. After reviewing the rubrics, this paper analyzes their validity and reliability for promoting the learning of neurophysiotherapy maneuvers and assessing the acquisition of the procedural skills they involve.

**Methods:**

In this cross-sectional and psychometric study, six experts and 142 undergraduate students of a neurophysiotherapy subject from a Spanish university participated. The rubrics’ validity (content and structural) and reliability (inter-rater and internal consistency) were analyzed. The students’ scores in the subject practical exam derived from the application of the rubrics, as well as the rubrics’ criteria difficulty and discrimination indices were also determined.

**Results:**

The rubrics´ content validity was found to be adequate (Content Validity Index > 0.90). These showed a unidimensional structure, and an acceptable internal consistency (α = 0.71) and inter-rater reliability (Fleiss’ ƙ=0.44, ICC = 0.94). The scores of the subject practical exam practically covered the entire range of possible theoretical scores, showing all the criterion medium-low to medium difficulty indices - except for the one related to the physical therapist position-. All the criterion exhibited adequate discrimination indices (rpbis > 0.39), as did the rubric as a whole (Ferguson’s δ = 0.86). Students highlighted the rubrics´ usefulness for learning the maneuvers, as well as their validity and reliability for formative and summative assessment.

**Conclusions:**

The changed rubrics constitute a valid and reliable instrument for evaluating the execution quality of neurophysiotherapy maneuvers from a summative evaluation viewpoint. This study facilitates the development of rubrics aimed at promoting different practical skills in health-science education.

**Supplementary Information:**

The online version contains supplementary material available at 10.1186/s40359-024-01643-7.

## Background

Procedural skills acquisition is essential for professional development in health science domains [1, 2], particularly in Physical Therapy, to prepare students for their clinical practice [3, 4]. Academically, these skills are usually acquired during undergraduate studies in laboratory classes [5], which are specialized practice rooms equipped as training facilities [2] where procedural skills are taught to a group of students. During these lessons, students simulate real-life clinical scenarios with each other or with mannequins [5–7]. They practice these skills in a secure and supportive environment under faculty supervision and receive regular feedback from both faculty and peers [8, 9]. Hence, in undergraduate studies, laboratory classes primary focus on mastering psychomotor skills, with less emphasis on clinical reasoning and the underlying knowledge of physiotherapeutic treatment interventions [2].

Teaching and training psychomotor skills and their practice are crucial for physical therapists, as hands-on clinical skills are one of their core competencies [2]. To achieve this, a traditional didactic [1, 2, 10] approach is used, following established concepts for instructing and acquiring clinical skills [5, 11, 12]. In this educational framework, a lecturer demonstrates a specific physiotherapeutic psychomotor skill or maneuver to students, while they observe the hands-on demonstration and listen to the lecturer’s explanations. Afterwards, students practice the demonstrated maneuver in pairs and receive feedback from either the lecturer or their peers. Then, this sequence is repeated with various maneuvers until the end of the lesson.

However, acquiring and applying these skills in a fluent, automatized, and contextualized manner is more complex than expected [13]. In Physical Therapy, their acquisition requires the development of a large number and diversity of learning practices [14], as well as frequent and abundant feedback focused on the levels of execution shown by the students [15]. But this is something hard to reach exclusively in university labs [16] since involves high personal and material resources, which are limited by time and location [6]. For this reason, providing instructional resources for students, increasing opportunities to practice beyond the classroom and promoting autonomous learning are essential [17]. This study was carried out under these circumstances. The study was conducted in a subject area that involved multiple practice groups. A total of 160 students divided into ten groups, who have only 30 h to learn a wide range of maneuvers related to neurophysiotherapy. These techniques include child physiotherapy and neuromotor development, proprioceptive neuromuscular facilitation, neurodevelopment treatment, orofacial dysfunction and physiotherapy for Parkinson’s disease and ataxia. Therefore, students require resources that allow them to practice maneuvers autonomously with peers outside the classroom and receive feedback tailored to their performance level, facilitating realistic self- and peer-assessment. In this regard, rubrics have already proven their usefulness. Previous research highlights that rubrics are especially useful tools to achieve complex practical skills; to frequently assess students with formative purposes as they provide high quality feedback and promote self-regulated learning (instructional or formative rubrics); to assess more objectively students’ performance on complex tasks; and to achieve higher consistency between raters (scoring or grading rubrics) [16, 18, 19].

In this respect, research with health sciences undergraduates (e.g. students of Medicine, Nursing, Psychology and Physical Therapy) has evidenced rubrics effectiveness in assessing and developing a wide range of competences (e.g. interpersonal communication and collaboration, and clinical case analysis), noting their increased consistency and reliable scoring ability and their positive effects on students’ learning outcomes [18, 20–23]. Different authors also emphasized the rubrics’ usefulness in integrating theoretical and practical training in the clinical formative process [24, 25]. Finally, rubrics can also address health-science students’ concern about the subjectivity of performance-based assessments and the inaccurate reflection of students’ performance, expressed in previous studies [24], guaranteeing justice in their evaluation. When rubrics are used, all students not only have the same conditions in the evaluation tests (time, space, available resources), but also in the application of the assessment criteria of their levels of performance [26].

However, evidence of the rubrics’ validity to facilitate the development of procedural skills in Physical Therapy is scarce, probably because the maneuvers exhibit high heterogeneity and specificity [20]. This is the case of the neuropathology clinical approach, which presents a complex learning challenge for students [27], as indicated by their feedback [15]. Thus, based on numerous pieces of evidence demonstrating the usefulness of rubrics in health sciences [18, 20–22], and addressing the complexity of dealing with large groups and the limited time available in class, the faculty of the neurophysiotherapy course of the Physical Therapy degree at the University of Valencia developed a set of rubrics to facilitate learning and evaluate the execution level of the maneuvers worked on in the course. Rubric development is a more time-consuming and complex process than other evaluation tools, but the benefits make rubrics a valuable addition to any course. This paper describes the psychometric validation process for the rubrics developed for training and grading purposes.

### Assessment rubrics: their usefulness for summative and formative assessment

Assessment rubrics have been a topic of special interest in educational psychology research in recent [18, 28, 29]. They are traditionally defined as “a coherent set of criteria for students’ work that includes descriptions of levels of performance quality on the criteria” [30]. Rubrics are primarily used for summative assessment [31, 32]. However, in recent years, their use has significantly increased not only from the perspective of summative assessment or as a grading tool, but also from a formative assessment perspective, guiding the learning and development of skills [18, 29, 33–35]. Thus, at present, a rubric can be defined as “a document that articulates the learning goals for a task and describes different levels of mastery of those goals” [36], which emphasizes their usefulness for both formative and summative purposes [28]. Thereby, research has primarily focused on analyzing their validity and reliability as assessment tools, with increasing attention being paid to analyzing their usefulness in improving learning outcomes, self-regulation, attitudes, and motivation toward student learning [28]. In any case, it is important to note that evaluation rubrics are tools that go beyond checklists, rating scales, or performance lists. They articulate expectations for student work by listing the criteria for the work and describing the levels of performance along a continuum of quality [37]. Thus, rubrics can be designed for both training and rating purposes, as performed in this study, by utilizing a table or matrix format that incorporates three essential elements: assessment criteria (five in total for this study), descriptions of quality/performance levels for each criterion and the corresponding levels to be attained (four levels in total for this study, ranging from “inadequate” to “advanced”), and a scoring strategy (criterion-based in this study, providing a pass/fail criterion and a total score for each maneuver execution).

Previous review studies on rubrics [18, 31, 32] emphasize that they were initially used mainly for summative purposes. These reviews indicate that rubrics can improve the reliable scoring of performance assessments, especially when they are analytic, topic-specific, and accompanied by exemplars and/or rater training. However, rubrics alone cannot guarantee valid assessment of performance. Incorporating a more comprehensive validity framework during the validation process could enhance the validity of the results [38–41]. This would not only improve psychometric properties, but also offer valuable insights into the effects on participants’ response processes, including the consequences on students’ learning outcomes and the ways in which rubrics are utilized and valued by teachers and students (e.g. students’ perceptions of their validity and usability for summative and/or formative purposes, their acceptance, fairness and justice in their application, the extent of faculty engagement and consistent use for feedback provision, or the availability of instructional time for this purpose). Additionally, as previously stated, rubrics have the potential to promote learning and/or enhance instructional quality.

In this line, previous research analyzing the usefulness of rubrics for summative purposes often assesses their content, external and/or construct validity, and sometimes reveals inadequate results [29, 31, 32, 42]. The content validity of rubrics is typically determined through expert judgment, with most studies demonstrating satisfactory results. External validity is typically assessed by correlating with other assessment instruments, with correlations coefficients typically ranging between 0.40 and 0.60 [32]. Structural validity is assessed through exploratory factor analysis techniques, which have been criticized in recent studies in favor of confirmatory factor analysis [43, 44], and/or through expert judgments on the alignment of guidelines, standards, and rubrics [45]. Numerous studies also analyze the intra- and inter-rater reliability of the rubrics’ application. Intra-rater reliability is typically assessed using Cronbach’s alpha, with most studies reporting values above 0.70 [46]. Several methods are used to assess inter-rater reliability [32], with numerous papers emphasizing the need of a trained assessor to apply rubrics consistently. These methods include: exact agreement between raters (range of values 55–75%), with 70% being the traditional criterion; the kappa statistic (range of values 0.20-0.63), with values between 0.40 and 0.75 representing fair agreement; and correlation of raters’ scores (range of values 0.27-0.98, with the majority between 0.55 and 0.75), with values above 0.70 considered acceptable. Finally, these studies also report educational consequences. They found that students and faculty tended to evaluate them positively. This was due to the fact that they make assessment criteria more explicit and clear, encourage reflective practice, provide faculty with information about the effectiveness of instructional practices, make it easier to provide students with higher quality feedback, or enable students to make more realistic self-assessments [15, 29].

In the latter line, although beyond the objectives of this study, research has increasingly focused on analyzing the usefulness of rubrics in promoting higher quality learning [29]. More specifically, a recent meta-analysis [28] demonstrated the positive impact of using rubrics on students’ academic performance, self-regulated learning, and self-efficacy for learning. The study also highlighted the moderating effect of educational level and the duration of the rubric intervention. The study’s results suggest that the use of rubrics has a positive moderating effect on academic achievement (*g* = 0.45, 95% CI [0.312, 0.831]). However, it also indicates a reduced positive effect on self-regulated learning (*g* = 0.23, 95% CI [-0.15, 0.60]) and self-efficacy (*g* = 0.18, 95% CI [-0.81, 0.91]). The positive effects on academic outcomes are evident across all educational levels, although younger students may require more extensive interventions. On the other hand, studies in higher education often report positive results from using rubrics, regardless of the length of the intervention [28, 37].

In any case, it is important to acknowledge the validity and reliability of rubrics in supporting valid conclusions about student learning, despite the emphasis on their use and formative utility [29, 42]. More specifically, among other criticisms, it is worth noting that various studies conclude that rubrics developed in higher education sometimes lack adequate alignment between criteria and learning goals [47–49], as well as adequate clarity and quality of language [18, 47, 50–52]. Likewise, studies have criticized rubrics for sharing explicit criteria with students, which may lead them to “meet the criteria” rather than deep learning [52, 53]. Another criticism relates to the lack of inter-rater reliability assessment to ensure that the criteria are applied uniformly, and that the scores provided are not affected by mood, fatigue, or implicit bias [54]. Numerous studies have also found that while students tend to view rubrics as learning tools, lecturers often prefer to use rubrics as a means of assigning grades quickly, objectively, and accurately [53, 55, 56]. Therefore, it is important to emphasize research that reconciles these two perspectives [36]. Finally, it should be noted that previous research cannot definitively determine the impact of rubric use on academic outcomes due to limited analysis of moderating variables such as gender differences, number of assessment criteria, performance levels, and timing of rubric use. This is due to the small number of previous studies, the need for more complex research designs, and the limited information provided about the characteristics, development process, and use of the rubrics [28] – please refer to additional file 1 for data related to the rubric characteristics used in this work. In summary, rubrics can promote learning, effective peer- and self-assessment, and even self-regulated learning, while streamlining the grading process and indicating whether and where instruction fell short, under the right conditions [18, 28, 29, 36, 42]. However, further research is necessary to gain a deeper understanding of this topic.

This section cannot be concluded without emphasizing that, in scientific literature, rubrics are often confused with other assessment tools, such as checklists, rating scales, or performance lists [28, 30, 37]. As an example, various work-based assessments (WBAs), such as the Mini-Clinical Evaluation Exercise (Mini-CEX) and Direct Observation of Procedural Skills (DOPS), have been widely used in health sciences to evaluate and provide feedback on trainees’ clinical skills [57]. Mini-CEX and DOPS require direct observation of trainees’ performance followed by a structured feedback conversation [58, 59] to improve their learning and clinical performance [60]. DOPS are mainly used to assess and facilitate the learning of procedural skills, while Mini-CEX are used primarily to promote clinical competences, both of which are used primarily in residency programs [61–63] and, to a much lesser extent, at the undergraduate level [64–68].

Although rubrics share training and grading purposes with WBAs, there are several differences between them. One particularly relevant difference for this study is that WBAs are typically used for direct observation of trainees’ performance with real patients and provide individualized face-to-face feedback to the trainee, making them more common in residency or postgraduate programs. In contrast, rubrics are more commonly utilized in undergraduate programs, as is the case in this study, which focuses on a third-year course. In this context, the number of students per group is much higher compared to residency programs. Time and resources for training are limited, especially in the case of neurophysiotherapy. Additionally, students lack prior knowledge and experience and typically only practice in simulated environments during laboratory classes, which are designed to acquire general and basic competencies and skills. Rubrics can be especially helpful in these circumstances compared to other evaluation tools. They promote the learning process of the skills involved and their self-regulation by specifying the steps and criteria to follow (which coincides with checklists, rating lists or the previously highlighted WBAs). Rubrics accurately describe the quality levels and standards to be achieved and the rating strategy to follow. In other words, receiving personalized feedback from faculty is crucial. However, in undergraduate studies, time constraints limit the availability of feedback compared to residency programs. Hence, rubrics enable autonomous practice learning outside the classroom by increasing the sources of feedback provision (including self- and peer-assessment) as they provide much clearer information about the standards to be achieved.

Another key distinction between WBAs and rubrics is that WBAs typically have more specific evaluation criteria. They are designed to assess and provide feedback to trainee practitioners on their proficiency levels in executing complex procedures and clinical skills across a wide variety of scenarios and patients, ensuring a sufficiently broad range of practice. Thus, complex procedures are often described in great detail and precision, but without a clear indication of the required level of proficiency. In the following structured formative evaluation, experts and residents analyze areas for improvement and establish action plans together. Rubrics, on the other hand, provide an explicit description of different levels of quality in the application of the criteria for satisfactory task development. They also specify the level considered adequate for each criterion and the grading strategy to assess their execution levels (apart from the fact that the rubrics developed in this work also include the steps to follow for executing each maneuver and the most common mistakes made in learning them). In summary, WBAs are intended to aid instructors in providing feedback that is tailored to the student’s level of performance in applying complex procedures, particularly in the workplace. Rubrics, on the other hand, make the criteria and performance levels more visible to students. This transparency allows for more realistic self- and peer-assessment [69], which facilitates the development of autonomous practices in the application of procedures. This is particularly important in the context and educational level of this study, as it extends beyond mere classroom practice and feedback received from faculty.

### Elaboration, use and evolution of the rubrics of neurophysiotherapy

Given that rubrics have shown usefulness in facilitating grading assessment and formative assessment, professors began developing and using rubrics during the 2017-18 academic year. The rubrics’ content relates to proprioceptive neuromuscular facilitation and neurodevelopment treatment because of their broad applicability in neurological diseases and their relevance in activating weakened muscles to promote greater participation in transfers among individuals with these pathologies [70]. The members of the educational innovation group signing this paper, made up of neurophysiotherapy faculty and an expert in educational psychology, developed the rubrics following the principles highlighted in research for the development of formative rubrics and good practices in their use [29, 49, 69, 71].

Although rubrics require a more time-consuming and complex preparation process compared to other evaluation tools, they can be useful in facilitating grading assessment and formative assessment in health sciences. Specially, their ability to facilitate the development of self- and peer-assessment of procedure execution levels is noteworthy. Rubrics help in the development of quality criteria and provide a description of the quality levels and common errors committed by undergraduates when learning them. As a result, after carefully analyzing the tasks involved in each maneuver, a detailed sequence to be followed in the maneuvers’ execution was introduced in the rubrics. This serves as an instructional guide for their application (please, refer to the additional file 2 for an example of the developed rubrics).

More specifically, we chose to develop assessment rubrics in the format mentioned earlier because we considered that they have numerous formative advantages over other evaluation instruments used in undergraduate studies. Rubrics specify to students the quality criteria considered in performing the tasks (five criteria in this case). The criteria provide a detailed qualitative description of different levels of performance (four levels - “inadequate”, “needs improvement”, “adequate” and “advanced” - scored 0, 1, 2 and 3 points, respectively), and the standard to be achieved in each criterion (“adequate” level, which reflects the achievement of mastery of performance). Rubrics present a criterion-based assessment strategy that is easy for students to comprehend. This strategy requires achieving an “adequate” performance level in each criterion. Additionally, a global numerical assessment is provided by calculating the sum of the scores obtained in the different criteria, with a minimum requirement of 10 points to pass and a maximum score of 15 points. This approach assists individuals in developing realistic expectations for performance levels and the distance they need to achieve beyond the minimum standards. This, in turn, promotes self-regulation to meet these expectations [31]. Furthermore, it enables faculty to provide higher quality feedback and allows students to conduct more realistic self- and peer-assessment to monitor their progress [18]. All of these characteristics mean that rubrics can be considered particularly suitable for formative assessment, as they focus on the process rather than the final learning outcome [15].

In the initial development process of the rubrics, as described in greater detail in a previous work [15], it was agreed (a) to consensually develop analytical rubrics for the 32 maneuvers considered in the subject; (b) to integrate identical criteria, assessment levels and grading strategy, as well as a verbal guide to support the execution, for each maneuver; (c) to distribute the responsibility for elaborating the rubrics’ initial version according to the teaching staff specialization, and subsequently discuss them until a consensus was reached on their final version; (d) to perform the rubrics’ review by teachers from other clinical specialties and seven students from previous courses, to verify their ease of understanding and evaluate their usefulness for formative and summative assessment; (e) to verify the consistency in their application by the teaching staff. The initial rubrics were perceived by students as valid and useful for learning, but the analysis of the final grades highlighted the need to improve their validity and reliability (e.g. internal consistency) [15].

Consequently, the faculty proposed modifications to optimize the rubrics’ usefulness, especially for scoring. Firstly, they agreed to maintain five criteria to evaluate the maneuvers, but replaced the previous criterion *fluency* (ability for performing the maneuver with ease and grace) -showed very limited variability and discrimination capacity- with a new one related to *holds* (support given by the physical therapist´s hands). Secondly, they agreed to describe in a clearer and more differentiated and exhaustive way the quality levels for each criterion incorporating additionally the most common errors committed by students in each maneuver. Hence, the rubrics’ new version was used in the 2020-21 academic year. It included five execution criteria with four quality levels and an execution support guide for each technique (an example is shown in the additional file 2). The modification procedure involved multiple meetings with the faculty in order to reach a consensus. More specifically, modifications were only adopted if the group’s agreement level was above 80%, which is the traditionally recommended percentage used in the modified Delphi method [72, 73].

The rubrics were used as a basic reference throughout the progress of the course in the 2020-21 academic year: (a) to facilitate learning the maneuvers both in the laboratory classroom sessions of the course and when students practiced autonomously, (b) to serve as a tool and basic reference for the formative assessment and to provide feedback to the students, (c) to promote self-regulated learning, as well as to perform self- and peer-assessment, (d) to use them as grading tools in the final test of the course -summative assessment. This paper analyzes the validity and reliability of the new version of the rubrics, examining if the changes introduced made it possible to overcome the limitations found in previous academic years, and expanding the evidence of validity and reliability thereof (e.g. structural validity, inter-rater reliability), which has been highlighted as particularly relevant in previous research [16, 18, 19].

This study presents relevant contributions to research on the usefulness of rubrics in health science education, particularly in undergraduate studies in Physical Therapy. Few studies have analyzed its usefulness in this area, especially from both a grading [21, 22, 74, 75] and formative evaluation perspective [15, 34]. This is even more relevant in the field of neurophysiotherapy, where this type of studies on this topic are still limited [76, 77]. In addition, this paper focuses on analyzing the rubrics’ usefulness in evaluating and facilitating learning of procedural skills. These abilities have been scarcely addressed in this research field given their high quantity and specificity [78]. Finally, this work exemplifies the continuous improvement process of this type of assessment tools to promote a better learning process and a more objective evaluation [18].

### Study aims and hypothesis

Rubrics have been shown to be useful in promoting learning and objectively grading student performance in various university tasks, particularly in health sciences studies [34, 74, 77, 79, 80]. However, previous research emphasizes the need to analyze their validity and reliability, especially in the field of neurophysiotherapy and in relation to learning and assessing procedural skills since few studies have been conducted. Therefore, the objectives of this work are as follows:

1. To determine the content validity of the rubrics developed through the judgment of experts in clinical and teaching neurophysiotherapy with extensive clinical experience in this field, and through the face validity of the students who use them.
2. To analyze the construct validity of the rubrics developed through confirmatory factor analysis techniques, hypothesizing that the five criteria considered (physical therapist position, patient position, verbal facilitation, holds and execution) are indicators of a single latent dimension related to the quality and degree of mastery in the execution of the maneuvers, as well as their internal consistency and inter-rater reliability.
3. To analyze the descriptive results of the scores obtained by students through the application of the assessment rubrics in the final practical exam of the subject, giving special importance to the number of students who passed/failed it according to the criteria established as passed, as well as the difficulty and discrimination indexes both of the individual criteria and of the rubrics considered as a whole.
4. To analyze the students’ assessment of the validity/reliability of the rubrics for grading the level of mastery of the maneuvers, as well as their utility/use for learning them.

## Methods

### Study design and participants

A cross-sectional study was performed during the 2020-21 academic year at the Faculty of Physiotherapy, University of Valencia (Valencia, Spain). Of the 160 students registered for the first time in the subject Physiotherapy in Clinical Specialties IV of the Physical Therapy Degree, 142 students (88.75% of students, average age 22.1 ± 12.1 years, 53.5% men) and six experts with wide clinical expertise in neurophysiotherapy participated voluntarily. Approval of the authors´ Institutional Ethics Committee was guaranteed (Code H1543332503311) and participants were informed about the study aims and their informed consent was obtained. This study complies with the Declaration of Helsinki.

In order to conduct the study with a statistical power of 95% (1 − β = 0.95), an a priori estimation of the minimum sample size required for structural equations models was made [63, 81, 82], establishing the statistical inference of the Type I error rate at the conventional limit (α = 0.05) and a small–medium effect size (*f* = 0.19) [83]. The results recommended that a minimum of 125 participants should be included in the sample, which is less than the number of students who participated in this study.

### Context of the study

In Physical Therapy, specialization only occurs at the master’s or postgraduate level, while at the undergraduate level, students acquire general competencies and skills. In Spain, the regulation for the Physical Therapy study program requires undergraduate students to engage in clinical practice with real patients during the second half of the program, specifically in the third and fourth years. At the University of Valencia, during the first year of clinical practices the focus is on musculoskeletal conditions. In the second year, the focus shifts to other specialties such as neurology, cardiorespiratory, pediatric, pelvic floor…, in accordance with the study plan. As a result, in the neurophysiotherapy course, taught in the second semester of the third year, undergraduates do not have clinical experience with neurological patients and only practice in simulation environments during laboratory classes.

### Procedures

The validity and reliability of a set of 32 rubrics (the rubrics´ tittles are shown in the additional file 3) were analyzed to determine the quality of the students´ maneuvers executions in the practical exam (grading rubrics), and to determine the students´ perception of the rubrics´ usefulness in facilitating learning (formative rubrics).

To determine the content validity of the rubrics, the modified Delphi method was applied [84, 85]. Six experts with extensive clinical and teaching experience in neurophysiotherapy from the local community initially completed an online questionnaire. The questionnaire aimed to assess their degree of agreement about the relevance, comprehensiveness and comprehensibility of the criteria considered, as well as the description of the levels of quality and standards to be achieved in the different criteria of the different maneuvers. Each panelist specified their suggestions for potential changes (round 1). This information was subsequently distributed and discussed through various face-to-face discussion groups (round 2) until a consensus was reached on the rubrics’ final wording, considering as a criterion that at least 80% of the experts expressed agreement with the adequacy of the rubrics [72, 73]. The students’ perception of the rubrics was also considered since the face validity of assessment instruments can affect their use and acceptability [18, 19, 86, 87]. After explaining and modeling students on how to use the rubrics, they completed an online questionnaire similar to the experts’.

To achieve valid scores from the rubrics’ application, they have to be reliable, which requires that several raters provide scores consistent with each other [18]. With this objective, and as recommended by previous research, the professors developed a rubrics’ consensus-driven norming or calibrating process workshop [88]. It consisted of three working sessions with structured discussions about the one-by-one application of each rubric criterion in the assessment of a video-recorded execution of three different maneuvers performed by students of preceding courses. Inter-rater reliability was determined by a follow-up exercise in which the professors independently rated the quality of the execution of nine video-recorded maneuvers, different from those considered in the norming workshop.

The rubrics´ internal consistency and structural validity, as well as the descriptive statistics of the scores derived from their application were determined by recording and analyzing the students’ scores obtained in the subject final practical exam. After receiving their results at the end of the academic year, the students completed an additional questionnaire on their perception of the rubrics’ validity and usefulness in evaluating their level of execution (summative evaluation) and in facilitating learning the maneuvers (formative evaluation).

### Measures

*Assessment rubrics*: They include five complementary criteria for grading the quality of the maneuver execution (physical therapist position, patient position, verbal facilitation, holds and execution) with the description of four performance levels for each criterion (0 = inadequate, 1 = needs improvement, 2 = adequate, 3 = advanced). The sum of the scores of each criterion provides the global score for the maneuver execution (0–15 points). The minimum competence, or basic level of proficiency or ability required to perform a given maneuver, was defined as obtaining an “adequate” performance level in all the criteria (a score of 10 points), or achieving this minimum overall score even if the “needs improvement” level was reached in any criterion, thus allowing for compensation (but not if an “inadequate” performance level was reached in any criterion). Thus, an overall score of less than 10 points is considered bellow basic or inadequate (fail) because the student demonstrates a level of proficiency or ability lower than the one required. A score of 10 or higher is considered adequate (pass), provided that no criterion is rated as inadequate. In the last case, three levels of proficiency can be achieved: basic or sufficient (10–11 points) in which the student demonstrates the required level of proficiency to execute the maneuvers; proficient or outstanding (12–13 points), which requires the achievement of an “advanced” rating in at least two of the criteria considered in the rubric; and advanced or excellent (A) (14–15 points), which requires achieving “advanced” rating in at least four of the quality criteria when executing the maneuvers, displaying a level of proficiency that exceeds the requirements to pass the course.

*Content validity questionnaires*: The experts´ questionnaire assessed, on a 4-level Likert-type response scale, the following: (a) the pertinence/necessity of each rubric criterion (1 = very low/not necessary, 4 = very high/essential); (b) its relevance/relative importance to assess the proficiency of the maneuver (1 = very unimportant, 4 = very important); (c) the adequacy/clarity of the descriptions provided for each criterion (1 = very inadequate, 4 = very adequate); and (d) the clarity to differentiate between quality levels of each criterion (1 = very unclear, 4 = very clear). The students’ questionnaire used the same response scales to assess: (a) the relevance/importance of each rubric criterion to learn the maneuvers; (b) the clarity of the descriptions provided for each criterion; and (c) the clarity to differentiate between quality levels in each criterion. In both questionnaires, there was an open question aimed at suggesting aspects to be modified in order to facilitate understanding and avoid ambiguities and misinterpretations.

*Quality of the maneuvers’ execution*: Scores obtained by students in the final practical exam (theoretical score range 0–15) calculated as the average of their execution levels in three different maneuvers assessed with the rubrics, were used.

*Rubrics’ validity and usefulness perception questionnaire*: This assessed the students’ perception of the rubrics’ validity/reliability (seven items) and utility/use for learning (10 items) on a 5-level Likert-type response scale (1 = totally disagree; 5 = totally agree). Both subscales showed high internal consistency (α = 0.93, in both cases).

### Statistical analyses

#### Content validity

The Content Validity Index (CVI) was used to determine the level of experts’ agreement on the relevance, the clarity/simplicity of the descriptions and the differentiation among the quality levels established in each criterion (CVI = number of experts agreeing on criteria rated as 3 or 4/total number of experts). The overall rubrics’ CVI (R-CVI) was calculated after determining the CVI for each specific criterion. The R-CVI was obtained by averaging the CVI of the different criteria for each maneuver. Also, using the same CVI formula, the face validity index (FVI) for each criterion and for the whole rubric (R-FVI) were determined from the students’ perceptions. CVI and FVI scores ≥0.80 were considered adequate for the different criteria [87, 89]. However, we consider a more restrictive value of 0.90 for R-CVI and R-FVI, following the recommendations of Polit and Beck [90], even though most studies consider 0.80 as satisfactory.

#### Construct validity

The rubrics’ structural validity was determined through confirmatory factor analysis using the Maximum Likelihood method for estimation. This approach was chosen because traditional exploratory factor analysis techniques have been widely criticized in health sciences education research [43]. Several goodness-of-fit indexes were considered, including Chi Square ratio, the Comparative Fit Index (CFI), the Non-Normed Fit Index (NNFI), the Root Mean Squared Error of Approximation (RMSEA), and the Standardized Root Mean Square Residual (SRMR). The cut-off criteria for reasonable fit were a CFI and NNFI of at least 0.90, RMSEA less than 0.06, and SRMR less than 0.08 [91].

#### Reliability

The internal consistency of the rubrics was assessed using Cronbach’s alpha, with a value of 0.70 or greater considered appropriate [92, 93]. Inter-rater reliability for each criterion was estimated by Fleiss’ kappa statistic (κ), and for the total score by the Intraclass Correlation Coefficient (ICC). κ values > 0.40 and ICC > 0.60 are considered adequate [18, 89, 94, 95].

#### Grade summary

Descriptive statistics of the students´ scores in the final practical exam (one-by-one criteria and rubric total score) were calculated. Also, the difficulty (p, percentage of correct executions) and discrimination (rpbis, point biseral correlation coefficient) indices for each criterion and for the total score (Ferguson’s delta, δ) were calculated [96].

#### Rubrics´ validity/reliability and utility/use perception

It was determined through the descriptive statistics of the students’ perceptions about both aspects.

Data analysis was conducted using SPSS statistical package version 28 (SPSS Inc., Chicago, IL), except for the Confirmatory Factor Analysis which was performed using the Statistical Package Eq. 6.1 [97].

## Results

### Content validity

The experts’ assessments highlighted the rubrics’ adequate content validity (Table 1). All of them considered the five rubrics’ criteria necessary and essential for proper assessment and scoring of maneuvers execution. Also, there was a high level of agreement among them regarding the studied variables, both in terms of individual criteria and when considering the rubrics as a whole (in all cases, CVI≥0.87, range 0.87 − 1.00).

**Table 1:** Experts rubrics’ content validity: consistency for criterion-level CVI and for rubrics’ level CVI (R-CVI)

Criterion	Relevance CVI	Clarity/simplicity CVI	Differentiation levels CVI
*0.- Execution support guide*	0.94	0.87	-
*1.- Physical therapist position*	0.98	0.98	1
*2.- Patient position*	1	1	1
*3.- Verbal facilitation*	0.96	0.98	0.94
*4.- Holds*	1	0.94	0.96
*5.- Execution*	1	0.94	0.94
*Rubric level index (R-CVI)*	0.99	0.97	0.97

CVI: Content Validity Index

The rubrics’ face validity was also very high (in all cases, FVI ≥0.89, range 0.89-0.98, Table 2), confirming that students mostly agreed that the criteria and the execution supporting guides were relevant/very relevant to facilitate learning (range 94–98%) and that provide clear/very clear (range 89–97%) and differentiated descriptions of their quality levels (range 92–98%).

**Table 2:** Students’ perceptions of the rubrics’ content

Criterion	Relevance FVI	Clarity/simplicity FVI	Differentiation levels FVI
*0.- Execution support guide*	0.95	0.92	-
*1.- Physical therapist position*	0.98	0.97	0.98
*2.- Patient position*	0.98	0.98	0.98
*3.- Verbal facilitation*	0.95	0.93	0.94
*4.- Holds*	0.94	0.89	0.92
*5.- Execution*	0.95	0.92	0.94
*Rubric level index (R-FVI)*	0.96	0.94	0.95

FVI: face Validity Index

Lastly, experts and students’ suggestions allowed to introduce improvements in the rubrics. For instance, in the maneuvers 23 and 24 (to know the exact technique, please see the additional file 3), the description and differentiation between levels of the holds, execution and verbal facilitation of the maneuver criteria were highlighted.

### Structural validity and reliability

Confirmatory Factor Analysis results highlighted that the hypothesized rubrics’ unidimensional model showed a satisfactory fit to the data (χ^2^/*df* = 1.540, *p* >.05; NNFI = 0.936; CFI = 0.981; SRMR = 0.032; RMSEA = 0.068, 95% CI [0.000-0.183]). All criteria also showed adequate standardized loadings, ranging from 0.42 (criterion 1, physical therapist position) to 0.65 (criterion 5, execution of the maneuver). The rubrics´ headings also revealed an acceptable internal consistency (α = 0.71, 95% CI [0.61-0.78]), all of them showing satisfactory homogeneity coefficients (Minimum = 0.38 for criteria 1; Maximum = 0.54 for criteria 5). Inter-rater reliability also resulted adequate when ratings were studied criteria-by-criteria and for the rubrics’ total score (κ = 0.435, 95% CI [0.432-0.438], *p* <.001; ICC = 0.943, 95% CI [0.823-0.986], *p* <.001).

### Grade summary

Table 3 presents the descriptive statistics of the students’ scores on the final practical exam, as well as the difficulty (p) and discrimination (rpbis) indices of each rubric criteria. The scores range (3–15) practically covered the entire range of theoretical scores (0–15), with a median of 10. Students were distributed among the four proficiency levels considered as follows: 45 (31.7%) failed to reach the minimum level required to demonstrate basic mastery of the maneuvers (bellow basic level); 46 students (32.4%) showed the required level of mastery (basic level) and obtained an overall score in the final practical exam equal to or higher than 10 and lower than 12 point; 32 students (22%) obtained an overall score in the final practical exam equal to or higher than 12 and lower than 14 points, which is considered a proficient level; and 19 students (13.4%) were classified as advanced, having scored 14 points or higher in the final practical exam, which indicates a very high level of mastery of the maneuvers required in the subject. Only two of them achieved the maximum score of 15 points.

**Table 3:** Students’ scores in the practical exam, level of difficulty and discrimination ability of the criterion

Criterion	M	SD	Mdn	Min	Max	Sk	Ku	p	rpbis
*1.- Physical therapist position*	2.69	0.65	3	0	3	-2.48	6.50	0.95	0.44
*2.- Patient position*	2.17	0.84	2	0	3	-0.69	-0.37	0.79	0.61
*3.- Verbal facilitation*	2.10	0.78	2	0	3	-0.20	-0.98	0.76	0.50
*4.- Holds*	1.73	1.08	2	0	3	-0.46	-1.06	0.67	0.65
*5.- Execution*	1.71	0.78	2	0	3	-0.38	-0.11	0.64	0.58
*Overall score*	10.40	2.79	10	3	15	-0.83	1.04	-	-

*M: Mean; SD:Standard deviation; Mdn: Median, Min: Minimum; Max: Maximum; Sk: Skewness; Ku: Kurtosis; p: difficulty index; rpbis: point biserial correlation coefficient*

Table 3 also shows that the criteria related to the physical therapist position presented the highest scores (Mdn = 3, range 0–3) and a very reduced difficulty index (*p* =.95). The rest of the criterion showed medium-low/medium levels of difficulty. All the criterion exhibited adequate discrimination coefficients (rpbis > 0.39), successfully differentiating between the students with the highest and the lowest scores. Lastly, the test as a whole (Ferguson’s δ = 0.86) also showed adequate discrimination levels [96].

### Students´ perception of the rubrics’ validity and usefulness

Students positively perceived the rubrics’ validity/reliability and utility/use. The highest rating items regarding validity/reliability deal with the integration of the most important elements to consider in the maneuvers and enabling the evaluation of the important skills in the subject (Table 4). The highest rating items regarding usefulness concern a better understanding of the assessment criteria and the easement of the maneuvers study/practice (Table 5). The rubrics’ perception of their validity/usefulness for the formative assessment and for the summative assessment was very similar (response scale range 0–10, average values of 7.91 and 8.06, respectively).

**Table 4:** Students´ perception of the rubrics´ validity/reliability

I think the rubric…	Mean	Standard Deviation	Range	Skewness	Kurtosis
1.- Integrates the most important elements to consider in the maneuvers	4.22	0.84	2–5	-1.2	1.5
2.- Makes it possible to evaluate the important competencies in this subject	4.17	1.00	1–5	-1.5	2.3
3.- Integrates criteria that will be useful to me in my future professional career	3.98	1.07	1–5	-1.0	0.5
4.- Is a reliable tool (makes it possible to measure the quality of the execution)	4.09	1.07	1–5	-1.2	1.1
5.- Fosters a fair comparison of the different students on the practical assessment test	4.00	1.23	1–5	-1.4	1.1
6.- Helps to understand the criteria involved in adequate performance	4.15	0.91	1–5	-1.7	3.7
*Overall average score*	4.10	0.87	1–5	-1.5	3.2

**Table 5:** Students´ perception of the rubrics´ utility and use

I think the rubric is useful for…	Mean	Standard Deviation	Range	Skewness	Kurtosis
1.- Clarifying how we have to perform each maneuver	3.98	0.90	1–5	-0.9	1.2
2.- Planning the study/practice of the maneuvers	3.72	1.01	1–5	-0.5	-0.3
3.- Reviewing what is learned in order to make adjustments	3.91	0.96	1–5	-0.9	0.6
4.- Realistically rating the execution of the maneuvers	3.87	0.99	1–5	-0.8	0.3
5.- Guiding the study/practice of the maneuvers	3.96	0.99	1–5	-1.0	0.7
6.- Discussing and determining what to improve in their execution	3.69	0.87	2–5	-0.2	-0.5
7.- Being able to perform the maneuvers with greater quality	3.93	0.94	2–5	-0.4	-0.8
8.- Facilitating the study/practice of the maneuvers	3.99	0.94	3–5	-0.7	-0.4
9.- Knowing more about the criteria that will be used to assess us	4.37	0.62	3–5	-0.5	-0.6
10.- Reducing my anxiety in the process of learning the maneuvers	3.67	1.09	1–5	-0.7	0.1
*Overall average score*	3.91	0.76	1.8-5.0	-0.6	0.3

## Discussion

The assessment of procedural skills represents an essential part of the formative process of health science professionals [78, 98]. Rubrics have been found particularly helpful in promoting students’ learning process (instructional rubrics) and determine their acquired level of competence (scoring rubrics) [18], as well as to integrate theoretical and practical knowledge of clinical competences [21, 25]. This perspective guided the development and use of a set of rubrics for the evaluation of neurophysiotherapy maneuvers in the Physical Therapy Degree. Intending to optimize their use for grading, various modifications were introduced in the 2020-21 academic year. They considered two basic findings of previous research: (a) analytic and topic-specific rubrics, completed with examples and/or rater training, can enhance the reliability of scoring when assessing performance [26]; and, (b) a comprehensive framework (validity) can facilitate valid judgement or performance assessment when validating a rubric [18, 32]. Thus, the criteria and quality levels for each maneuver were described more clearly and exhaustively, between-rater consistency was increased by developing a calibration workshop, and their validity and reliability evidences were expanded [16, 18, 32].

The content validity of the rubrics new version was analyzed by a panel of experts, finding that all the criteria were considered essential. Also, they showed very high levels of agreement concerning the relative importance, clarity/simplicity and differentiation between quality levels established in the rubrics (CVI ≥0.87, in all cases). The students’ perceptions confirmed the rubrics´ face validity and their adequate understanding (FVI ≥0.89, in all cases). Experts and students’ suggestions allowed introducing improvements to the rubrics to facilitate their understanding and avoid ambiguities and misinterpretations. Evidence highlights that the student involvement in rubrics´ design and implementation is critical for their success [19, 23].

To ensure valid rubrics’ scores, they must first be reliable. Therefore, it is essential to verify both the internal consistency and inter-rater reliability [18, 32, 94]. Results evidenced their adequate internal consistency (α = 0.71), noting that different criteria can be assumed to be interrelated and combined in a single overall score [87, 89]. The inter-rater reliability of the rubrics’ application also showed adequacy with satisfactory levels of consistency among raters in each individual criterion and in the total score [89, 95]. Its structural validity was also evidenced, confirming its unidimensionality [94]. In health science education, many concerns exist regarding the performance-based assessment of students [99]. Also, assessment still mainly relies on non-standardized methods [100]. Additionally, standardized scoring tools, like rubrics, also help protecting students’ mental well-being related to assessments by eliminating prejudice and personal bias of the examiners [101]. Hence, studies that validate assessment tools are crucial in this discipline.

The students’ final exam scores covered practically the entire theoretical range of possible scores. Their median (10) coincided with the score of a student achieving the adequate standard of each criterion. All criteria -except for the physical therapist position- showed medium to medium-low difficulty indices, and satisfactory discrimination coefficients, as did the total score [96]. In agreement with previous results [15, 23], students positively valued the rubrics´ validity/reliability and usefulness for learning, facilitating transparent evaluation criteria and allowing autonomous practice.

Competency-based education is at the forefront of health science education and procedural skills are fundamental to clinical practice [98]. In fact, insufficient training of procedural skills has been related to mistakes in health interventions [17]. However, there is little evidence of rubric’s validity to facilitate their achievement. In this regard, this study can serve to exemplify a method of development, validation and continuous improvement of rubrics aimed at enhancing the teaching-learning process of procedural skills in health sciences education. Moreover, it facilitates the development of new rubrics aimed at promoting different procedural skills by defining common quality criteria for executing basic procedural skills and maneuvers, and providing specific support guidelines for each one.

### Strengths, limitations and future research

The results of this study have numerous practical implications for using the developed rubrics to teach, learn, and assess basic procedural skills in neurophysiotherapy undergraduate studies. These implications go beyond determining the rubrics’ validity and reliability. They also focus on the rubrics’ usefulness for subject development and management of large groups of students, as well as their acceptability, fairness, and feasibility in application. In this sense, the most relevant implications of this work are:

1. The rubrics developed facilitate instructional alignment between learning objectives and outcomes to be achieved, teaching methodology and resources to be used during classes, and the methodology, tools and performance standards to be considered in the subject assessment exams. The coherence and integration of all components of instructional planning, including learning objectives, activities, content, resources, and assessment, are essential to help students achieve the intended learning outcomes. Lack of alignment between objectives, learning activities, and assessment is a much more common problem than it might seem in university contexts [102, 103].
2. The rubrics have facilitated alignment and congruence between formative and summative assessment. The faculty have used them to offer feedback to students on their performance levels throughout the course, and for grading in the final practical assessment. In any case, numerous studies highlight that rubrics are often viewed by professors as tools for objective grading, but with limited formative value [18, 31]. These studies also suggest that students have reported ambiguity regarding the purpose (formative or summative) of rubrics used by teachers or even instructors in the case of WBAs [58, 59]. In this study, the rubrics’ validity and utility for both summative and formative assessment were highly valued by the students. This result emphasizes the dual role of rubrics [28, 29, 31, 104] for formative (e.g., facilitating learning through constructive feedback provided by faculty and peers, and the possibility of carrying out abundant autonomous practice and more realistic self-assessment) and summative purposes (e.g., allowing to determine the skill level reached throughout the subject), and also other aspects such as their content validity or their usefulness for the sake of the students’ professional future. The dual role of rubrics, both formative and summative, an aspect to be considered in the use of all assessment instruments, is shared by the previously mentioned WBAs, which are primarily intended to provide feedback for directing training rather than for summative assessment.
3. From the beginning of the course, rubrics provide students with a comprehensive catalog of procedural skills to develop, steps to follow for maneuver execution (derived from task analyses performed during application), quality criteria and levels of mastery to achieve, and the most common mistakes made during learning. All of these characteristics help students develop a comprehensive understanding of the learning outcomes they need to achieve. This enables them to focus their attention and efforts on these outcomes and the general framework for evaluation, resulting in a greater likelihood of self-regulating the learning processes and achieving better academic results [28]. In this line, the neurophysiotherapy students have highlighted that the rubrics helped them to plan, monitor, and evaluate their learning processes more effectively, which emphasizes the transparency of the criteria and performance standards that must be met.
4. Rubrics enable faculty to provide feedback on students’ performance levels by using rubric-referenced verbal feedback in class. This allows for detailed and individualized information about students’ strengths and weaknesses to be provided in a manageable time frame [18, 29, 31, 105]. As pointed out by students themselves, these evaluation methods facilitate a more objective and fair assessment of the performance levels demonstrated in the practical exam. This study also shows adequate inter-rater reliability in the application of rubrics.
5. Additionally, rubrics enable students to complete a significant amount of practice work throughout the semester, beyond in-person practice with faculty support, as well as more realistic self- and peer- assessment. This allows students to receive ongoing feedback from multiple sources [18, 29, 31, 105]. This issue is especially relevant in undergraduate studies and even in later specialization studies or residency. Due to time constraints to develop psychomotor skills and large student groups, numerous autonomous practice opportunities to develop psychomotor skills is needed. These practices should include quality feedback tailored to the students’ performance levels that aligns with criteria and quality standards. Rubrics can provide quick and easy access to clear criteria for self-and peer-assessment [69, 104] in developing autonomous learning practices beyond the laboratory classroom.

However, it is important to acknowledge that this study has limitations that should be considered in future research. Firstly, the participants were limited to a single university, resulting in a relatively small sample. Nonetheless, it is worth noting that almost 90% of the students enrolled in the neurophysiotherapy subject of the Physical Therapy degree participated in the study. Future studies should consider larger and more representative samples, including participants from different universities to ensure greater generalization of results. In this regard, it is important to account for significant differences between universities in terms of available resources, the possibility of practicing with neurological patients, the number of students per group, the extension of subjects and their location in the curricula. To ensure a comprehensive understanding of the topic, it is important to consider these facts regarding Physical Therapy studies in our context, as well as the diversity of clinical practices in different clinical specialties.

Future studies should also analyze the validity and usefulness of the rubrics in other levels and training contexts, such as postgraduate or specialization studies, and with practicing professionals, using real patients in clinical settings. It would be worthwhile to analyze the extent to which mastery of maneuvers learned in the subject is maintained during clinical internships with patients in the following course, which is the final year of the degree program, and its predictive capacity on learning outcomes. These questions could also be addressed in postgraduate studies. Research has repeatedly shown that procedural skills decay after extended periods of nonuse [106]. The retention of these skills is most affected by the teaching methodology used (e.g. instructional methods, degree of overlearning, and assessment criteria), the trainee’s ability and motivation, and task-related aspects (e.g. length of the retention interval between learning and application, difficulty and complexity, speed and accuracy requirements). Furthermore, it is important to note that clinical practice for both undergraduate and postgraduate students involves working with real patients who have diverse characteristics, and require the efficient integration of a wide range of maneuvers. Thus, it is essential to assess and optimize the maintenance of basic skills and sub-skills when planning future training. In this regard, it would be valuable to investigate the impact of practicing basic subskills in simulated patients on the preparation and utilization in subsequent clinical practice, as well as readiness to begin postgraduate studies. However, this question would require extending the available instructional time. In any case, simulation training could be systematically integrated into routine procedural training [107] in undergraduate Physical Therapy studies before working with real patients (this issue is much more noticeable in Medicine and Nursing studies in our context). This would promote the acquisition of basic skills and their integration into more complex procedures that respond to the most common and habitual situations in professional practice [108, 109].

Secondly, a more complex research design could have broadened the conclusions and practical implications of this work. A longitudinal study with multiple formative assessments conducted by different evaluators, which is characteristic of competence-based training [20, 86, 110, 111], would have allowed for the assessment and monitor of learning curves for maneuvers. This would have provided a greater volume of validity indicators for the rubrics by empirically determining the progress and performance levels that students would have been achieving until the pre-specified quality standards were met [112]. Although there is no consensus on the best methodology [113], the Cumulative Sum curve (CUSUM) is one of the most frequently used methods for evaluating psychomotor education in healthcare [114, 115]. CUSUM offers advanced statistical techniques to assess trainees’ performance evolution and detect significant changes in their learning process over time. It takes into account the success rate of task performance, the evaluation method’s failure possibilities, and acceptable/unacceptable failure probability [113, 116]. These techniques are commonly used in specialized medical training to monitor the learning of complex technical procedures, such as mastery of noninvasive surgical techniques. This study addresses less complex procedures at lower training levels and over shorter periods of time. Nevertheless, their consideration would be particularly useful in studies conducted at advanced levels of specialization in neurophysiotherapy. This would enable the identification of the adequacy of the rhythms and levels of learning that trainees achieve throughout the training process regarding the application of the maneuvers in patients with varying types and levels of affectation. This issue has not yet been routinely incorporated into the CUSUM models [113]. On the other hand, these studies would also allow for the determination of the predictive validity of rubrics. This would involve determining whether undergraduate students’ mastery of basic maneuvers can shorten the learning curve at more advanced levels.

Thirdly, although the methodology and research design align with those typically used in this type of validation study, it would have been beneficial to employ more robust designs. This would facilitate not only the analysis of the progression and pace of learning the maneuvers throughout the subject (already highlighted in the preceding paragraph), but also the quality of self-assessment conducted by the participants and the comparison of the potential positive learning effects of rubrics versus other resources. To achieve this, the levels of execution should be evaluated at different times (at least, before and after the subject and during a follow-up period) and/or various experimental conditions should be considered (for example, intervention 1 using rubrics, intervention 2 using modeling, supervised practice and feedback in the classroom, and intervention 3 receiving additional intervention with rubrics and/or other training resources such as instructional videos). In this line, it would be interesting to compare the acceptability, feasibility, and instructional efficacy of the developed rubrics with other types of assessment tools previously highlighted and used in postgraduate training (e.g. DOPS and Mini-CEX) or in combination with them. Recent meta-analytical studies have shown that assessment rubrics have a positive effect on learning levels in university studies [28], as well as that the Mini-CEX and DOPS are useful for promoting learning among postgraduate medical trainees [60, 117]. In agreement with these authors, we would like to emphasize the presence of various factors that can enhance or diminish the effectiveness of these methods for training physicians. They include organizational culture (e.g. the value of teaching and feedback), work structure (e.g. the time available for application and faculty development), instruments (e.g. the content of assessment), and users (e.g. the relationship between trainees and supervisors). These considerations highlight important aspects to be addressed in research on training in health sciences. Future research should aim to answer not only which tools are more effective, but also determine the conditions, training levels, available resources, learning outcomes, learning contents to be addressed, use of them and the essential purpose of the formative and/or summative process.

## Conclusions

The developed rubrics constitute a valid and reliable instrument for evaluating the execution quality of neurophysiotherapy maneuvers (summative evaluation). Physical Therapy students also highlight that rubrics are very useful for the summative (scoring rubrics) and formative (instructional rubrics) assessment, emphasizing their acceptability, fairness and feasibility in their application. Thus, the developed rubrics are especially useful for improving and aligning the teaching, learning and assessment of procedural skills; for providing students with greater opportunities to practice and perform more realistic self-assessment; and for enabling faculty to evaluate the potential impact of incorporating new teaching strategies and instructional resources on their students’ learning outcomes. Finally, this study facilitates the development of rubrics aimed at promoting different procedural skills in health-sciences University teaching by defining common quality criteria for their execution and providing specific support guidelines for each practical maneuver.

### Electronic supplementary material

Below is the link to the electronic supplementary material.

Supplementary Material 1: Additional file 1.docx

Supplementary Material 2: Additional file 2.docx

Supplementary Material 3: Additional file 3.docx

## Data Availability

The datasets used and/or analyzed during the current study are available from the corresponding author on reasonable request.

## Abbreviations

95% CI: 95% confidence interval
Bartlett: Bartlett’s test of sphericity
CFI: Comparative Fit Index
CUSUM: Cumulative Sum curve
CVI: Content Validity Index
DOPS: Direct Observation of Procedural Skills
FVI: face validity index
ICC: Intraclass Correlation Coefficient
Mini-CEX: Mini-Clinical Evaluation Exercise
NNFI: Non-Normed Fit Index
p: percentage of correct executions
R-CVI: Content Validity Index of the rubric as a whole
R-FVI: face validity index of the rubric as a whole
RMSEA: Root Mean Squared Error of Approximation
rpbis: point biseral correlation coefficient
SRMR: Standardized Root Mean Square Residual
WBAs: work-based assessments
