# Supplementary material for: Improving assessment of procedural skills in health sciences education: a validation study of a rubrics system in neurophysiotherapy
Source: BMC Psychol. 2024 Mar 14;12:147. doi: 10.1186/s40359-024-01643-7 (PMC10941460; doi:10.1186/s40359-024-01643-7)
Supplement: Supplementary file 1 — Supplementary Material 1: Additional file 1.docx [file 40359_2024_1643_MOESM1_ESM.docx]

**Instrument to report the characteristics of rubric design and implementation**

Created by Panadero, E., Jonsson, A., Pinedo, L. & Fernández-Castilla, B (2023)*.* Effects of Rubrics on Academic Performance, Self-Regulated Learning, and self-Efficacy: a Meta-analytic Review*. Educational Psychology Review*.

| IMPROVING ASSESSMENT OF PROCEDURAL SKILLS IN HEALTH SCIENCES EDUCATION: A VALIDATION STUDY OF A RUBRICS SYSTEM IN NEUROPHYSIOTHERAPY  Authors:  Rafael García-Ros, Maria-Arantzazu Ruescas-Nicolau, Natalia Cezón-Serrano, Cristina Flor-Rufino, Constanza San Martin-Valenzuela and M. Luz Sánchez-Sánchez  **Our study investigates:**  Rubrics and scoring accuracy  Rubrics and academic performance  Rubrics and students’ perceptions  Rubrics and_________________________________________________________________________________ |
| --- |

**Describe the characteristics of your rubric intervention study in the table below.**

| **Design** | | | |
| --- | --- | --- | --- |
|  | Category | Description | Our study |
| **1** | **Rubric presence** | Have you included the rubric in the publication as supplementary material? | Yes  No. Reason: Click here to add text |
| **2** | **Assessment criteria** | Number of assessment criteria included in the rubric | 5 |
| **3** | **Performance levels** | How many performance levels are included in the rubric? Also list the headings | 4 Inadequate, needs improvement, adequate and advanced |
| **4** | **Creation** | Was the rubric created for this study? If not, please indicate the original source | Yes  No |
| **5** | **Scoring strategy** | If the rubric contains an explicit scoring strategy, provide a brief description. | Addition of all the criteria scores. To pass the execution, an “adequate” performance level must be obtained in all criteria (total score of 10 points), or if a lower performance level (basic) is obtained in any criteria, the total score must still reach 10 points. |
| **6** | **Type** | How was the assessment communicated to the students, holistic (i.e., as an overall assessment for all criteria or  analytical (i.e., separately for all criteria assessed)? | Holistic  Analytical |
| **7** | **Type 2** | Was the rubric general (i.e., a general skill such as writing), task-generic (i.e., applicable to several similar tasks) or  task-specific (i.e., only applicable to one particular task) | General  Task-generic  Task-specific |
| **Implementation** | | | |
| **8** | **Self-assessment** | Was the rubric used for self-assessment? | Yes  No |
| **9** | **Self-scoring** | Was the rubric used to calculate a self-score? | Yes, but the self-score was not included in the final grade.  Yes, and the self-score represented _% of the final grade.  No |
| **10** | **Peer assessment** | Was the rubric used for peer assessment? | Yes  No |
| **11** | **Peer score** | Was the rubric used to score a peer? | Yes, but the peer score was not included in the final grade.  Yes, and the peer score represented __% of the final grade.  No |
| **12** | **Feedback** | Did the students receive additional feedback about their performance or on how they used the rubric? | Yes, on both  Only on their performance  Only on how they used the rubric  No  If yes, could you describe the additional feedback characteristics?  Elaborated Informative-Corrective Feedback |
| **13** | **Official weight** | Did the activity assessed with the rubric count towards the students' grade? | Yes, for a 100 % of the total  No  Se utiliza en la prueba de evaluación ifnal |
| **14** | **Frequency** | How many times was the rubric used? (Once, twice, etc.) | In both all the face-to-face practical sessions carried out in the subject, and during autonomous learning outside the classroom as a self- and peer-assessment tool. |
| **15** | **Training** | Did the participants receive training about the rubric? If yes, describe the training and the specific moment in which they received it. | Yes. Explanatory videos on its structure and use. Explanation and modeling of its use in class. Discussion and feedback on its use. |
| **16** | **Revision** | Did learners revise their work after using the rubric? | No  Yes |
| **17** | **Extent of involvement** | How were learners involved in the rubric design and implementation? | Students just received and used the rubric  Students were allowed to make small changes to the rubrics  Students made substantial changes  Students co-created the rubric  Other: Clarity and appropriateness of the language used, differentiation between levels in the criteria |
| **18** | **Use of other instruments** | Were any additional instruments employed to further strengthen the intervention effects, or to make comparisons with the rubric? If so, please, explain the characteristics of those instruments | Integration with video modeling |
| **19** | **Technology** | Was any type of technology used for the design and/or the implementation of the rubric? If so, please provide the details | No |
| **Outcomes** | | | |
| **19** | **Study Outcomes** | These variables are directly measured as outcomes of the rubric activity.  Select all the options that apply to your study from the right column. | **Beliefs & perceptions:** including perceptions of learning capacity to use the rubric (e.g., fairness, usefulness), metacognition and self-regulation, attitudes and beliefs (e.g., self-efficacy), teachers’ perceptions/conceptions.  **Emotions and motivation:** emotions experienced by learners (e.g., achievement emotions, social emotions, etc.) & motivational beliefs (e.g., learning motivation).  **Performance:** academic/domain specific performance, achievement, improved draft/work (i.e., revision).  **Skills:** quality of contribution to the group, professional behaviour, problem solving skills, work habits, interpersonal skills, metacognitive & self-regulatory skills.  **Reliability of rubric:** consistency of rubric scores among different raters (e.g., several teachers).  **Validity of** **rubric:** aspects related to testing the validity, such as content validity, comparing students and teachers' assessment, etc.  **Other:** Click here to add text |
| **Moderators/mediators** | | | |
| **20** | **Moderators/mediators** | Variables that are not usually manipulated but are taken into account when investigating rubrics. Select the variables that have been explored in your study from the right column. | **Gender:** of assessor/assessee.  **Ability & Skills:** includes prior knowledge, prior performance, achievement level, GPA, finished high school, previous level of education, year of enrolment, etc.  **Skills:** reviewing ability, computer skills, etc.  **Age/grade level:** of assessor/assessee.  **Other:** Click here to add text |
| The design of this tool is based on an instrument to report peer assessment design characteristics from: Panadero, E., Alqassab, M., Fernández Ruiz, J., & Ocampo, J. C. (2023). A systematic review on peer assessment: Intrapersonal and interpersonal factors. *Assessment & Evaluation In Higher Education*, 1-23. <https://doi.org/10.1080/02602938.2023.2164884>.  The two last categories (19 and 20) based on Alqassab, M., Strijbos, J., Panadero, E., Fernández Ruiz, J., Warren, M., & To, J. (2023). A systematic review of peer assessment design elements. *Educational Psychology Review*.  <https://doi.org/10.1007/s10648-023-09723-7>  If your intervention included peer assessment, we recommend you also fill out that instrument and include it as a supplementary material in your publication. It can be found here: <https://osf.io/5k42z/?view_only=c77740eca9ef44978e1ac47abcaeef7c> | | | |
