# Supplementary material for: Improving assessment of procedural skills in health sciences education: a validation study of a rubrics system in neurophysiotherapy
Source: BMC Psychol. 2024 Mar 14;12:147. doi: 10.1186/s40359-024-01643-7 (PMC10941460; doi:10.1186/s40359-024-01643-7)
Supplement: Supplementary file 2 — Supplementary Material 2: Additional file 2.docx [file 40359_2024_1643_MOESM2_ESM.docx]

**Additional file 1.** Example of a rubric of a neurodevelopmental treatment maneuver

| **No. 5. Change from the postural set of sitting to the postural set of supine** | | | | | | | | | |
| --- | --- | --- | --- | --- | --- | --- | --- | --- | --- |
| Starting from the correct postural set of sitting (see rubric N.6), the PhT raises the stretcher to the level of his greater trochanter and stands on the patient's paretic side. | **The PhT facilitates movement from the PT's CKP, placing the caudal hand in front and the cranial hand behind.** | **The PhT creates a forward rotational movement of the CKP, flexing the trunk of the PT while facilitating the rotation of the trunk towards the non-paretic side.** | From the caudal hand (located previously on the CKP), the PhT lowers the trunk of the PT onto the stretcher. | **With the elbow of the cranial hand, the PhT keeps the PT's head in slight flexion throughout the descencing movement to the stretcher (avoiding hyperextension).** | At the last moment, before the PT reaches the stretcher, the PhT brings the PT's trunk towards himself/herself, placing it parallel to the stretcher, making it easier for the PT to finish lifting the non-paretic LL on the stretcher. | The PhT lets the PT's head fall gently onto the pillows, without the head hitting in extension. | **With the caudal hand, the PhT grabs the foot of the PT in neutral dorsiflexion and with the cranial hand stabilizes the most affected knee at the same time that asks the PT to raise the paretic LL onto the stretcher.** | The PT is placed in the appropriate postural set of supine by means of the bridge maneuver (see rubric Nº3). | The technique is performed fluently |

| **RUBRIC:** | | | | |
| --- | --- | --- | --- | --- |
|  | **INADEQUATE (0)** | **NEEDS IMPROVEMENT (1)** | **ADEQUATE (2)** | **ADVANCED (3)** |
| **Physiotherapist position** | The PhT is not on the proper side of the stretcher and the height of the stretcher is also incorrect. | The PhT is not on the proper side of the stretcher but the height of the stretcher is correct. | The PhT is on the proper side of the stretcher, but the height of the stretcher is not correct. | The PhT is on the proper side of the stretcher and the height of the stretcher is correct. |
| **Position of the subject with hemiparesis** | The subject starts in an incorrect postural set of sitting and ends in an incorrect postural set of supine. | The subject starts and ends in postural sets that are practically correct (in the absence of one or two components). | The subject starts and ends in correctly postural sets but the student does not know the reason for them. | The subject starts and ends in correct postural sets and the student knows the reason for them. |
| **Verbal facilitation of the maneuver** | The subject's voluntary activity is not facilitated (not indicating the maneuver to be carried out, nor the activity to be carried out). | The subject's voluntary activity is scarcely facilitated since only some activity to be carried out is indicated. | The subject's voluntary activity is mostly facilitated but it is forgotten to indicate one or two activities to be carried out. | The subject's voluntary activity is totally facilitated, always indicating the maneuver and the activity to be carried out. |
| **Holds** | The holds are not correct (PhT grasps the paretic ankle or wrist, instead of holding the paretic foot and hand in a functional position).  ***Common Mistake: Improper CKP hold and/or improper head hold.*** | The holds are not correct (PhT grasps the paretic ankle or wrist, instead of holding the paretic foot and hand in a functional position). | The holds are correct but the PhT does not know the reason for them and/or shows some error in handling during the execution. | Automation of the correct holds is demonstrated; i.e. manipulating the subject is carried out with the perfect holds and knowing the reason for them. |
| **Execution of the maneuver** | The PhT does not identify the maneuver or performs the sequence of the maneuver inadequately.  ***Common error: a) The PhT does not protect the paretic shoulder (PhT does not place the paretic UL outside the stretcher so that it remains free and the subject does not lean on it while descending the body); b) The movement of the CKP is not facilitated correctly (PhT does not facilitate trunk flexion or rotation).*** | The sequence of the maneuver is not completely correct.  ***Common error: a) PhT scarcely protects the paretic shoulder (the paretic UL is placed outside the stretcher but during the maneuver it does not remain free and the subject leans slightly on it while descending the body); b) PhT hardly facilitates the movement of the CKP (facilitates trunk flexion but not rotation).***  **ASSESSMENT REQUIREMENT ≥1** | The PhT performs the sequence of the maneuver properly but presents some carelessness in the execution.  ***Common mistake: a) PhT correctly protects the paretic shoulder (PhT places the paretic UL off the stretcher and during the maneuver it remains free and the subject does not lean on it while descending the body), but without knowing the reason); b) PhT correctly facilitates the movement of the CKP (facilitates trunk flexion and rotation, but without knowing the reason).*** | The manipulation of the subject is perfect and PhT performs properly and fluently the sequence of the maneuver.  The PhT correctly protects the paretic shoulder and facilitates the movement of the CKP knowing the reason for both. |

PhT: Physical therapist, PT: patient, CKP: central key point, LL: lower limb, UL: upper limb.
