# Supplementary material for: Improving assessment of procedural skills in health sciences education: a validation study of a rubrics system in neurophysiotherapy
Source: BMC Psychol. 2024 Mar 14;12:147. doi: 10.1186/s40359-024-01643-7 (PMC10941460; doi:10.1186/s40359-024-01643-7)
Supplement: Supplementary file 3 — Supplementary Material 3: Additional file 3.docx [file 40359_2024_1643_MOESM3_ESM.docx]

**Additional file 2.** Title list of the validated rubrics

| **Proprioceptive Neuromuscular Facilitation** |
| --- |
| 1. Scapula: anterior elevation-posterior depression pattern  2. Scapula: anterior depression-posterior elevation pattern  3. Pelvis: anterior elevation-posterior depression pattern  4. Pelvis: anterior depression -posterior elevation pattern  5. Upper extremity: flexion-adduction-external rotation pattern  6. Upper extremity: extension-abduction-internal rotation pattern  7. Upper extremity: flexion-abduction-external rotation pattern  8. Upper extremity: extension-adduction-internal rotation pattern  9. Lower extremity: flexion-adduction-external rotation pattern  10. Lower extremity: extension-abduction-internal rotation pattern  11. Lower extremity: flexion-abduction-internal rotation pattern  12. Lower extremity: extension-adduction-external rotation pattern  13. Upper trunk stabilisation: Agonist reversal  14. Upper trunk stabilisation: Combined isotonic technique  15. Upper trunk rhythmic stabilization |
| **Neurodevelopment treatment** |
| 16.- Change from the postural set of supine to the postural set of side lying on the non-paretic hemibody  17.- Change from the postural set of supine to the postural set of side lying on the paretic hemibody  18.- Mobility in supine: Bridge exercise  19.- Change from the postural set of supine to the postural set of sitting  20.- Change from the postural set of sitting to the postural set of supine  21.- Moving from the postural set of sitting to the postural set of standing with anterior support  22.- Activation of proximal analytical mobility of the paretic upper limb  23.- Activation of analytical mobility of the forearm and hand of the paretic upper limb  24.- Facilitation of the reciprocal innervation of both hemipelvis  25.- Righting reactions outside the midline  26.- Triceps surae tone modulation manoeuvre  27.- Moving from sitting to standing from the key point of the pelvis  28.- Body weight transfer to the paretic hemibody in standing  29.- Standing prone  30.- Gait re-education: swing phase  31.- Gait re-education: stance phase  32.- Facilitation of walking from the central key point and the pelvis key point |
